# Illusory Stimuli Can Be Used to Identify Retinal Blind Spots

**DOI:** 10.1371/journal.pone.0001060

**Published:** 2007-10-24

**Authors:** Michael D. Crossland, Steven C. Dakin, Peter J. Bex

**Affiliations:** 1 University College London (UCL) Institute of Ophthalmology, London, United Kingdom; 2 Schepens Eye Research Institute, Harvard University, Cambridge, Massachusetts, United States of America; University of Minnesota, United States of America

## Abstract

**Background:**

Identification of visual field loss in people with retinal disease is not straightforward as people with eye disease are frequently unaware of substantial deficits in their visual field, as a consequence of perceptual completion (“filling-in”) of affected areas.

**Methodology:**

We attempted to induce a compelling visual illusion known as the *induced twinkle after-effect (TwAE)* in eight patients with retinal scotomas. Half of these patients experience filling-in of their scotomas such that they are unaware of the presence of their scotoma, and conventional campimetric techniques can not be used to identify their vision loss. The region of the TwAE was compared to microperimetry maps of the retinal lesion.

**Principal Findings:**

Six of our eight participants experienced the TwAE. This effect occurred in three of the four people who filled-in their scotoma. The boundary of the TwAE showed good agreement with the boundary of lesion, as determined by microperimetry.

**Conclusion:**

For the first time, we have determined vision loss by asking patients to report the presence of an illusory percept in blind areas, rather than the absence of a real stimulus. This illusory technique is quick, accurate and not subject to the effects of filling-in.

## Introduction

The induced twinkle after-effect (TwAE), first described by Ramachandran and Gregory in 1991, is a compelling illusion observed in normally sighted subjects who are deprived of visual input to a restricted area of retina using an artificial scotoma [1], [2]. Subjects adapt to a dynamic noise stimulus containing a small mean luminance region (the artificial scotoma). When the adaptation pattern is replaced with a uniform screen, the area that contained noise appears blank, while the area formerly occupied by the artificial scotoma appears to contain “twinkling” noise: the TwAE.

The locus of the TwAE within the visual pathway is not known. Ramachandran and Gregory proposed that “whatever mechanism is responsible for this induction of twinkle…is unlikely to be very different from the process causing the filling in of the scotoma in the first place” [1]. Electrophysiological and imaging studies indicate that filling in is likely to be mediated by higher level cortical areas [3]–[5]. Hardage and Tyler identified several key differences between filling-in and the TwAE including the larger area over which the TwAE can be observed (at least 20°, compared to 1.5° for filling-in), the absence of any chromatic component to the TwAE, and the absence of the TwAE when the temporal frequency of the dynamic noise is below 10Hz [6], [7]. Functional MRI experiments add weight to their hypothesis that the effect is mediated by an inhibitory rebound of high level large receptive fields rather than a persistence of filling in [8].

The most common cause of retinal scotomas, and indeed the principal cause of blindness, in Europe and the USA is age-related macular disease (AMD)[9], [10]. In advanced AMD central visual field is lost through an atrophic or neovascular process [11]. Whilst atrophic forms of macular disease remain untreatable, angiostatic and antiangiogenic agents can be used to retard the development of neovascularisation in AMD [12], [13]. The gold standard technique for identifying scotomas in people with AMD is retinal specific microperimetry: a time consuming procedure requiring the skilled use of specialised equipment limited to few clinical or research centres. Conventional perimetric tests, whilst more widely available, are not appropriate due to the poor fixation stability [14], [15] and noncentral fixation locus [16]–[18] of many people with retinal disease. Campimetric techniques involve patients identifying regions of absence within a homogenous field [19]–[21], but perceptual completion may limit the ability of many patients to identify visual field defects using this technique. Those at risk of scotoma development are given a simple grid chart to observe on a daily or weekly basis [22]: however this technique has poor sensitivity, again due to perceptual completion over the scotoma [23]–[26].

**Figure 1:**
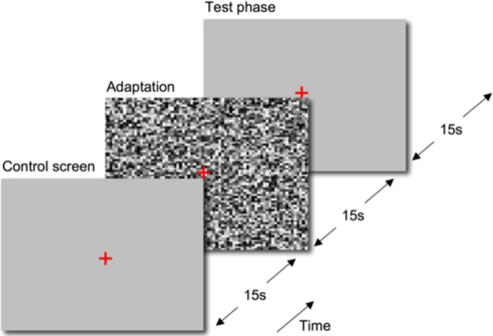
Stimulus presentation. The *control screen* was an isoluminant grey screen presented for 15 seconds to determine whether subjects could trace their scotoma boundary against a regular background. The *adaptation phase* was a dynamic field of random greyscale noise presented at 60Hz for 15 seconds. The *test phase* was a further isoluminant grey screen presented for 15 seconds. Subjects were asked to mark the boundary of any illusory area by means of a touchscreen mounted to the monitor. A red cross of arm length 1° was present throughout to guide fixation for the drawing phase. Adaptation and test phase were repeated to a total of five times.

Given the difficulty of identifying retinal scotomas in people who fill-in their blind spot, we sought to determine whether the TwAE might be adapted to identify scotomas in people with macular disease. To this end, we identified eight patients with central and paracentral scotomas caused by macular disease. The location, shape and extent of these scotomas was carefully mapped with retinal specific microperimetery. Next we had subjects view a large field of dynamic (60Hz) noise followed by a homogenous grey test screen in thirty second cycles (figure 1). Patients were asked to fixate a cross that was continuously present, although eye movements during the noise phase were unimportant since the stimulus was a uniform, spatially extensive noise field. During the test phases, subjects were asked to identify areas of any perceived anomaly in the blank screen by tracing around them with a stylus on a touch-sensitive screen mounted over the computer monitor. All participants were unfamiliar with the TwAE before the study and were asked to describe the appearance of the adapting and test screens.

## Results

Four of the eight subjects reported that the noise pattern appeared to fill in across their scotoma. Six of the eight experienced a TwAE during the test phases and could easily trace around its perimeter. The effect was variously described as an “aberration”, a “twinkling”, or most descriptively, as a “moving cumulus cloud” (table 1). Of the four subjects who experienced filling-in (who would not respond to conventional campimetric tests), three experienced the TwAE.

**Table 1:** Patient characteristics and results

Initials	Age (years)	Diagnosis	Visual acuity (logMAR)	Filling in	Description of TwAE
PF	82	AMD:CNVM	0.92	No	‘aberration’
KJ	78	AMD:CNVM	1.50	Yes	No effect
SP	35	Traumatic maculopathy	0.32	No	‘twinkling’
EP		AMD:CNVM	0.82	No	‘dark patch’
TK	79	AMD: GA	0.30	Yes	‘mistiness’
GW	77	AMD: GA	1.36	Yes	‘cumulus cloud’
VA	69	AMD&IPCV	0.62	No	No effect
MK	76	AMD:CNVM&GA	1.06	Yes	‘grey shimmer’

Diagnosis and visual acuity are given for poorer eye. Filling in: Whether participant reports perceptual completion in everyday life. TwAE: Twinkle after-effect. AMD: Age-related macular disease. CNVM: choroidal neovascular membrane. GA: geographic atrophy. IPCV: Idiopathic polypoidal choroidal vasculopathy.

All subjects who experienced the TwAE were able to fully describe the boundary of their scotoma within the test phase. For the two subjects who did not experience the TwAE immediately, the size of each square element in the noise pattern was varied to have side of between 0.05° and 0.8°, and the stimuli were presented many times under these different conditions. Neither subject reported any TwAE even after these manipulations.

**Figure 2:**
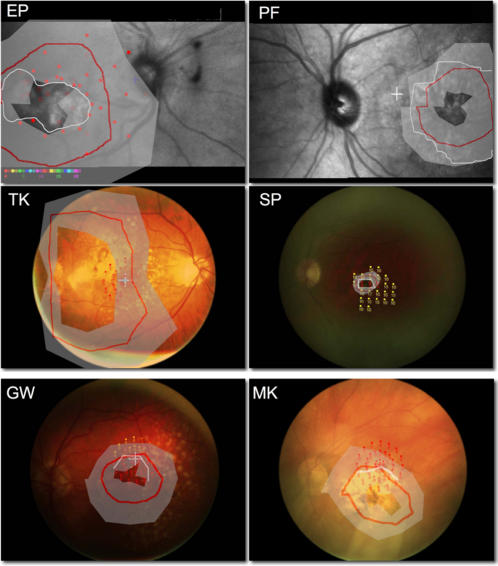
Scotomas identified by the dynamic after effect. Red line represents mean scotoma boundary; grey region indicates 95% confidence interval of estimate. White line represents boundary of dense scotoma identified from microperimetry assessment (except subject TK where scotoma is larger than the area measured using microperimetry, and GW and MK where only the upper boundary of the scotoma could be identified). Where present, white cross indicates centre of fixation. EP, PF: microperimetry performed using Rodenstock SLO-101 scanning laser ophthalmoscope. TK, SP, GW, MK: microperimetry performed using Nidek MP-1 microperimeter.

Figure 2 compares the location of visual field loss defined by microperimetry with the location of the TwAE mapped by the observers. The white lines show the boundary of scotomas defined by microperimetry. The red lines show the geometric mean of the apparent perimeter of the TwAE mapped by the observers. The shaded grey areas indicate 95% confidence intervals on this mean, based on a minimum of five repetitions. There was excellent agreement between both measures of the scotoma.

## Discussion

We have demonstrated that the induced TwAE can be used to map retinal scotomas accurately in some patients with central and paracentral scotomas arising from macular disease. Our findings build on earlier anecdotal reports of the TwAE in patients with retinal scotomas [2], [27], and provide the first quantification of the TwAE in patients with eye disease. We believe that this is the first time patients have been asked to report the presence of an illusory stimulus, rather than the absence of a real stimulus, to determine the area of non-seeing retina.

Although it is difficult to determine exactly what participants perceived during the test phase, their eloquent and unprompted descriptions (table 1) indicate that they were experiencing the TwAE. No participants perceived any irregularity on a uniform grey field without the period of adaptation, indicating that the effect described was not simple mapping of the apparent scotoma boundary. As the adaptation and test stimuli were of equal mean luminance, it is unlikely that patients were reporting a simple luminance afterimage during the test phase.

Three of our participants did not experience perceptual completion over their scotomas yet did experience a TwAE. This observation supports Hardage and Tyler's proposal that filling-in and the TwAE are complementary mechanisms. More encouragingly, three of our patients described the TwAE despite experiencing completion of the dynamic noise pattern and not responding to a standard campimetric test.

What is the mechanism driving this phenomenon? As the TwAE can not be induced at temporal frequencies of less than 10Hz, a location within the magnocellular pathway seems likely [7]. The large area over which the TwAE can be experienced (up to 20°) suggests that a likely locus would be a later area of the visual pathway such as the middle temporal complex (MT+) where neurones have very large receptive fields, frequently extending 10° into the ipsilateral visual field[28]. Further, MT neurons in the macaque have been shown to respond to stimuli presented well outside their classical receptive fields (by distances of >15°) even without stimulation within the receptive field[29]. An alternative candidate location is far earlier in the visual pathway: *y*-type retinal ganglion cells are known to have extensive horizontal connections and which respond preferentially to dynamic stimuli [30], [31].

**Figure 3:**
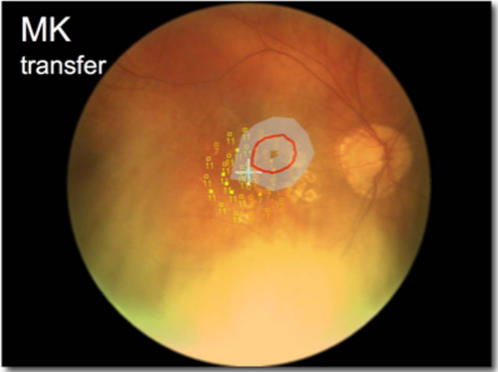
Transferred after image for subject MK. Red line indicates mean scotoma boundary assessed using dynamic after effect. Subject observed the adapting pattern with his left eye which has a dense central scotoma (figure 1) whilst covering the right eye. On a given signal MK moved his hand from his right to his left eye and observed the isoluminant screen. No dense scotoma was identified using microperimetry in the right eye but paracentral areas of relative scotoma were present.

Unlike retinal ganglion cells, MT+ neurons do not show any preference for the eye of stimulation, so should the effect be cortical in origin we would expect the TwAE to transfer between the eyes. Whilst Hardage and Tyler [6] found no interocular transfer of the TwAE in normally-sighted observers, recent work by Morgan and colleagues demonstrates interocular transfer of a facilitatory effect induced under similar circumstances to the TwAE [32]. To determine whether the effect could be transferred in patients with visual field loss, we performed further examination of one of our subjects who had highly incongruous scotomas: MK. We did not attempt to induce a transferred TwAE in any other observer. MK's right eye had small areas of paracentral scotoma caused by geographic atrophy, whereas his left eye had a large central scotoma. We presented the adapting stimulus to his poorer (left) eye whilst occluding the right eye. On a given signal, the test screen was displayed, the left eye was uncovered and the right eye was covered. MK then reported a robust TwAE, but only in the areas corresponding to the locations of scotoma in his left eye (figure 3).

The reason that MK experienced the induced twinkle only in the areas of scotoma in his left eye (and not within the region of scotoma in the right eye) may explain the lack of transfer experienced by control subjects. We speculate that rivalry is introduced between the salient test screen in one eye and the TwAE in the other. Such rivalry could suppress the presence of the TwAE in normally-sighted observers and limit inter-ocular transfer of the TwAE. However, patient MK's vision contains regions where no retinal input is present in the test eye: that is, within the scotoma. These regions of sighted and non-sighted vision in the test eye set up “piecemeal” rivalry across his visual field[33]. Under these conditions, perception of the test screen dominates in sighted areas of the test eye but the TwAE dominates perception in the non-sighted areas of the test eye. We conclude that the TwAE is likely to manifest within MT+.

It is not clear why two of our participants (KJ and VA) did not experience a TwAE. No apparent differences were identified in their scotoma properties, duration of disease, fixation locus or visual function. Nor was it related to the occurrence of filling in–one did and one did not experience filling-in. In order to determine whether visibility of the noise was the limiting factor, the size of the inducing noise stimulus was varied by a factor of 16: from squares of side 0.05° of visual angle to 0.8°. One explanation could be that these two patients were unable to divide their attention between the fixation point and the region of the TwAE. People with macular disease are known to adopt a preferred retinal locus (PRL) for fixation in peripheral retina and, in some respects, reference their eye movements to this “pseudofovea” [16], [17], [34]. It is not clear whether plasticity exists in the primary visual cortex in humans with macular disease [35], [36]. It is possible that different levels of cortical reorganisation had occurred in patients who did experience the TwAE compared to those who did not.

Unlike subjects with artificial scotomas [27] our patients did not systematically underreport the size of their scotoma. Although the 95% confidence interval on our estimates of scotoma size are large, it took under three minutes to perform five trials on the TwAE test, compared to around 20 minutes per eye for microperimetry. If determining scotoma size is critical then further repetition of our test would reduce the size of the confidence intervals. Alternatively, the TwAE test could be used as a screening test to identify scotomas prior to detailed microperimetric testing within this region. It should be noted that confidence limits of scotoma size are not produced by commercially available microperimeters, yet quantification of uncertainty in the measurement of the scotoma boundary are critical for assessing changes in scotoma area during disease progression or following treatment.

We do not aim to suggest that the limited number of subjects in this study is sufficient to imply that this type of test should be introduced into routine clinical practice: nor do we suggest that a test with sensitivity of 75% is suitable for screening for this condition. However, we feel that the concept of using illusory stimuli to determine the absence of function is appealing and novel. We suggest that similar illusory techniques could be used by colleagues in clinical ophthalmology and other areas of the cognitive neurosciences to determine and investigate the absence of function.

## Materials and Methods

All subjects had age-related macular disease causing a central scotoma diagnosed by a consultant ophthalmologist (other than SP who had a traumatic maculopathy caused by a road traffic accident). No subjects had any other eye disease or any history of neurological disease. All participants had binocular scotomas except SP, who had one eye enucleated in adulthood due to ocular trauma. The study was approved by the Camden&Islington PCT Local Research Ethics Committee of the UK National Health Service. Subjects gave their informed consent prior to data collection and the study conformed to the tenets of the Declaration of Helsinki.

### Microperimetry

Microperimetry was performed using either the SLO-101 Scanning laser ophthalmoscope (Rodenstock, Germany) or the Nidek MP-1 microperimeter (Nidek, Italy). In both cases, subjects were asked to observe a central fixation cross whilst retinal specific perimetry was performed of the central retina. Stimuli were Goldmann III targets presented against a dark background. In the MP-1, the automated 10-2 strategy was used to measure threshold retinal sensitivity. In the SLO, manual perimetry was performed at one intensity (200 cd/m^2^). In both cases microperimetry maps were automatically superimposed on the retinal image by tracking a retinal landmark.

### Determining the TwAE

Stimuli were created on an Apple computer using custom functions written in Matlab (v.7.3; Mathworks, Natick, MA) based on elements of the Psychophysics toolbox[37] ^,^ [38] and were presented on an 18” CRT monitor (Ultrascan P991; Dell, Round Rock, TX) with a 60Hz refresh rate. The peak screen luminance, measured using a photometer (Minolta CS-100, Konica Minolta, Japan) was 145 cd/m^2^ when viewed through the touch screen panel.

Subjects observed the stimuli monocularly with their better eye, whilst the contralateral eye was occluded with an opaque eye patch. Subjects sat 50cm from the computer screen. Appropriate refractive correction was worn using full-aperture trial lenses.

The experimental procedure is illustrated in Figure 1. First, a mean luminance grey screen was presented for a period of 15 seconds. Subjects were asked to identify any missing or irregular areas on the screen. Next, dynamic greyscale noise was presented for a period of 15 seconds. Each element of noise was square, of side 12 pixels (29 min arc) and was randomly assigned a new luminance value between 1 and 145 cd/m^2^ every 17 msec (60Hz). Finally, an isoluminant grey screen was presented for 15 seconds. Subjects were asked to draw around the edge of any area of irregularity with a stylus using a touchscreen (MagicTouch KTMT-1921; Keytec Inc., Garland, TX). Screen coordinates of the stylus location were recorded at 60Hz. A central fixation cross of arm length 1° was displayed throughout, and subjects were asked to maintain fixation on this point. The adaptation and test phase was repeated to a total of five presentations.

Additional testing for inter-ocular transfer of the TwAE was performed on subject MK. He viewed the 15 sec dynamic noise stimulus with his poorer (left) eye. At the onset of the adapting noise, an audible stimulus was heard, the test screen was displayed, his left eye was uncovered and his right eye was occluded. As before the subject was asked to indicate any anomalous areas of the display by marking the touch screen as above.

All data were analysed with Matlab. The Cartesian pixel coordinates of cursor position were converted to polar values with respect to the centre of the drawn area for each trial. The orientation of each co-ordinate (*θ*) was rounded into 30° bins from 0 deg. The mean distance from the centre and standard deviation (*σ*) of that value were calculated for each of the 12 polar coordinates. A dodecagon was constructed for the mean and mean ±1.96σ of the data at each coordinate. Using Photoshop (v.7.0; Adobe, San Jose, CA) these dodecagons were flipped vertically to correctly represent visual field space. For each subject, the dodecagons were resized to the retinal image obtained by the microperimeter/SLO by equating 5° of visual space to the horizontal extent of the optic disc[39]. The resized dodecagons were superimposed on the fundus image. For clarity, the dodecagon representing the mean position was drawn in red, and the area between the 5% and 95% dodecagons was shaded grey.
